# Radiofrequency Ablation of Liver VX2 Tumor: Experimental Results with MR Diffusion-Weighted Imaging at 3.0T

**DOI:** 10.1371/journal.pone.0104239

**Published:** 2014-08-07

**Authors:** Yubao Liu, Ligong Lu, Haosheng Jin, Xiaoming Chen, Zhonglin Zhang, Zaiyi Liu, Changhong Liang

**Affiliations:** 1 Department of Radiology, Guangdong General Hospital, Guangdong Academy of Medical Sciences, Guangzhou, Guangdong, the People's Republic of China; 2 Department of Interventional Therapy, Guangdong General Hospital, Guangdong Academy of Medical Sciences, Guangzhou, Guangdong, the People's Republic of China; 3 Department of General Surgery, Guangdong General Hospital, Guangdong Academy of Medical Sciences, Guangzhou, Guangdong, the People's Republic of China; Northwestern University Feinberg School of Medicine, United States of America

## Abstract

**Purpose:**

To evaluate the value of DWI in detecting the lesions of pre- and post-radiofrequency ablation (RFA) of the rabbit liver VX2 tumors.

**Materials and Methods:**

Twenty-two New Zealand White rabbits were tested. The protocol was approved by the Committee on the Ethics of Animal Experiments. Twenty separate tumor fragments were implanted into the livers of 20 rabbits, the liver was exposed by performing midline laparotomy. 3.0T MR DWI (b = 0, 200, 400, 600, 800,1000 s/mm^2^) were performed 14–21 days after tumor implantation (mean, 17 days) in the 18 tumor-bearing animals. Then RFA was performed in the 18 tumor-bearing animals and in the two healthy animals. 3.0T MR DWI was performed 7–10 days after RFA (mean, 8 days). Pathology exam was performed immediately after the completion of post- RFA MR imaging. Analyzing the features of MRI and ADC values in the pre- and post- RFA lesions of the VX2 tumors, and histopathologic results were compared with imaging findings.

**Results:**

The difference of ADC value between viable tumor and normal liver parenchyma was significant (*P*<.001). After RFA, when b = 200, 400, 600, 800, 1000 s/mm^2^, the differences of ADC values of viable tumor, granulation tissue, necrosis, normal liver parenchyma were significant (*P*<.001). At the time the animals were sacrificed after RFA and MR imaging, histopathologic results of local viable tumors were found in 9 (50%) of the 18 treated tumors. Macroscopic viable tumors were found at the RFA sites in 3 (17%), all 3 macroscopic viable tumors were visualized at the periphery of the RFA areas.

**Conclusions:**

3.0T MR DWI can be used to follow up the progress of the RFA lesion, it is useful in detecting different tissues after RFA, and it is valuable in the further clinical research.

## Introduction

Radiofrequency ablation (RFA) has been widely used as a desirable treatment method for hepatic tumors. RFA techniques are attractive because they are amenable to minimally invasive approaches, as compared with resection [1], [2]. Imaging is important for procedural guidance and for precise assessment of treatment adequacy [3]. An understanding of normal and abnormal imaging characteristics is needed to assess treatment adequacy.

Conventional magnetic resonance imaging (MRI) cannot provide exact information about the features of the tissues after RFA therapy. Some imaging features of the tissues after RFA such as post-treatment granulation tissue can be similar to that of viable tumor, which makes early detection of residual or recurrent tumor at the RFA site difficult [4], [5].

It is impossible to precisely correlate imaging findings with local tumor recurrence in clinical patients because complete histopathologic exam is not available. So imaging features of recurrent or residual tumor after RFA are poorly understood. Previously validated procedures for DWI of VX2 rabbit liver tumors showed that DWI can distinguish viable from necrotic tumor, however, previous imaged tumors had not been treated by RFA [6], [7]. Few studies are available about 3.0T MR DWI in evaluating hepatic lesions after RFA.

The purpose of this study was to evaluate the values of 3.0T MR DWI in assessing the rabbit liver VX2 tumor before and after RFA, and to detect the correlation between 3.0T MR DWI findings with histopathologic results.

## Materials and Methods

### 1. Animal and Tumor Model

Twenty-two New Zealand White rabbits weighting 2.5–3.5 kg were tested. The protocol was approved by the Committee on the Ethics of Animal Experiments of Guangdong General Hospital, Guangdong Academy of Medical Sciences (Permit Number: GDREC2013091A), and this study was carried out in strict accordance with the recommendations in the Guide for the Care and Use of Laboratory Animals of the National Institutes of Health. All surgery was performed under sodium pentobarbital anesthesia, and all efforts were made to minimize suffering. Experiments were performed in accordance with institutional guidelines. Prior to all procedures, including tumor implantation, RFA, and imaging, all rabbits were fasted for 4–6 hours with free access to water, and received an intramuscular injection of 1.0 mg/kg of body weight of acepromazine maleate and 35 mg/kg of ketamine hydrochloride. Intravenous access was then acquired via marginal ear vein.

The VX2 tumor strain was transplanted into the hind thigh muscles of tumor carrier rabbits. The tumor carrier rabbit was killed, and then the VX2 tumor was excised and placed in the saline. Necrotic tissue was discarded and the tumor tissue was cut into 1 mm^3^ fragments. The liver was exposed by performing midline laparotomy, twenty separate tumor fragments were implanted into the livers of 20 rabbits. Two normal rabbits were used as control group for RFA of the normal liver.

The rabbits were sacrificed after the last post procedure MR scan using an overdose of IV thiopental sodium, and the liver was harvested for histologic analysis.

Fourteen to 21 d (mean 17 d) after tumor implantation, MRI T1WI, T2WI, CT plain scan examinations were performed in all rabbits. Lesions with one of the following features were eligible for the inclusion criteria: the lesion located within the liver parenchyma; the lesion measured less than 3 cm in maximum diameter; the area of necrosis less than half of the lesion in size. Lesions with one of the following features were excluded from the study: the lesion extended to surface of the liver, measured more than 3 cm in maximum diameter, or with an area of necrosis more than half of the lesion.

### 2. RFA Procedure

All the 18 tumor-bearing animals and the two healthy animals underwent RFA. All percutaneous procedures of RFA were performed under CT scan guidance. The radiofrequency system was RITA medical systems 1500. The power of the generator was set at 150 W and maintained for 6–8 minutes, the tip temperature maintained at 75∼90°C. After successfully puncturing the electrode into the liver via a small incision of the skin, then correcting its position and direction according to CT images, and the ideal position of the electrode was the center of the lesion or approaching to the center of the lesion.

### 3. MR DWI protocol

All rabbits underwent MRI and DWI exams in 7–10 days after RFA (mean, 8 days). All images were acquired in supine position after anesthesia. T1WI, T2WI, gadolinium-enhanced T1WI, and DWI(TR 1200 ms, TE 45.1/56.1/60.1/68.2/75.5 ms corresponding to b values: 0, 200, 400, 600, 800, 1000 s/mm^2^) were used 14–21 days after tumor implantation (mean, 17 days) in the 18 tumor-bearing animals by 3.0T MR scanner (Signa Excite; GE Medical Systems, Milwaukee, WI) using cylindrical knee radiofrequency coil. Slice thickness: 3∼5 mm, matrix: 128×128, FOV 24 cm×24 cm, NEX = 4. Gadolinium-enhanced T1WI was acquired after 0.1 mmol/kg body weight gadopentetate dimeglumine (Magnevist; Bayer, Berlin, Germany) was administered to each animal via the ear vein by means of manual fast bolus injection. The ADC map was generated from b-values of 0 and 200 s/mm^2^, 0 and 400 s/mm^2^, 0 and 600 s/mm^2^, 0 and 800 s/mm^2^, 0 and 1000 s/mm^2^ with the monoexponential fit.

### 4. Image and pathologic analysis

Two radiologists interpreted MR images by consensus without being provided with any pathologic information. Imaging outcomes included tumor size, presence of viable tumor tissue, necrotic tissue, normal tissue and granulation tissue. Image interpretation was performed with conventional sequence images and DWI. Pathology was performed immediately after the completion of post-RFA MR imaging. Measuring the ADC values of pre- and post-RFA lesions of VX2 tumors and normal rabbits, analyzing the features of MRI and ADC values on the pre- and post-RFA images, and histopathologic results were compared with imaging findings.

DWI was manually delineated at a GE AW4.3 FuncTool Performance workstation with dedicated software (GE Medical Systems). For ADC measurement, an ellipsoid region-of-interest (ROI) of approximately 10–30 mm^2^ was drawn on index DW image (b = 0 s/mm^2^), and these ROIs were subsequently copied onto ADC map. The means of the intensities of the ROI for the lesions were expressed as relative mean intensities automatically converted to ADC. All ADC values were expressed as mean ± standard deviation (SD).

### 5. Statistical analysis

Repeated measurement data analysis of variance (ANOVA) was applied to compare parameters, followed by least significant difference (LSD) tests for multiple comparisons of quantitative data. *P*<.05 was considered statistically significant.

## Results

All the 20 rabbit liver models of VX2 tumors were constructed successfully, one died because of anesthetized overdose, another one was excluded due to the lesion extending to the surface of the liver. All 18 untreated VX2 tumors displayed remarkable hypointensities or isointensities on T1-weighted images, hyperintensities or isointensities on T2-weighted images, and heterogeneous enhancements on contrast enhancement images.

Eighteen liver VX2 tumors measured 8–25 mm in diameter (mean diameter, 15 mm), and all were found during laparotomy of RFA procedures. The tumors and 2 normal rabbit livers were treated with RFA. No severe complications appeared except 2 suffered mild burns on the back skin.

**Figure 1:**
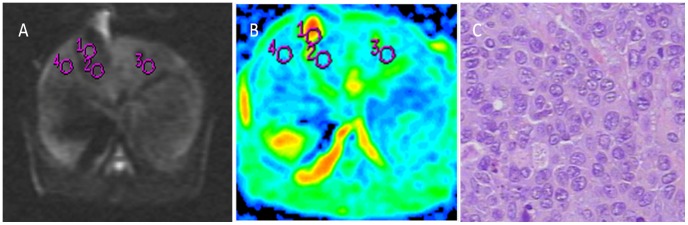
MRI of rabbit liver 8 d after RFA. DWI (A) of rabbit liver, hypointensity appeared in necrotic lesion, nodular lesion of hyperintensity around the necrotic lesion. DWI ADC map (B), area 1 referred to viable tumor. Pathology of area 1 (C). HE×200, viable tumor was consistent with area 1 of (A), (B).

**Figure 2:**
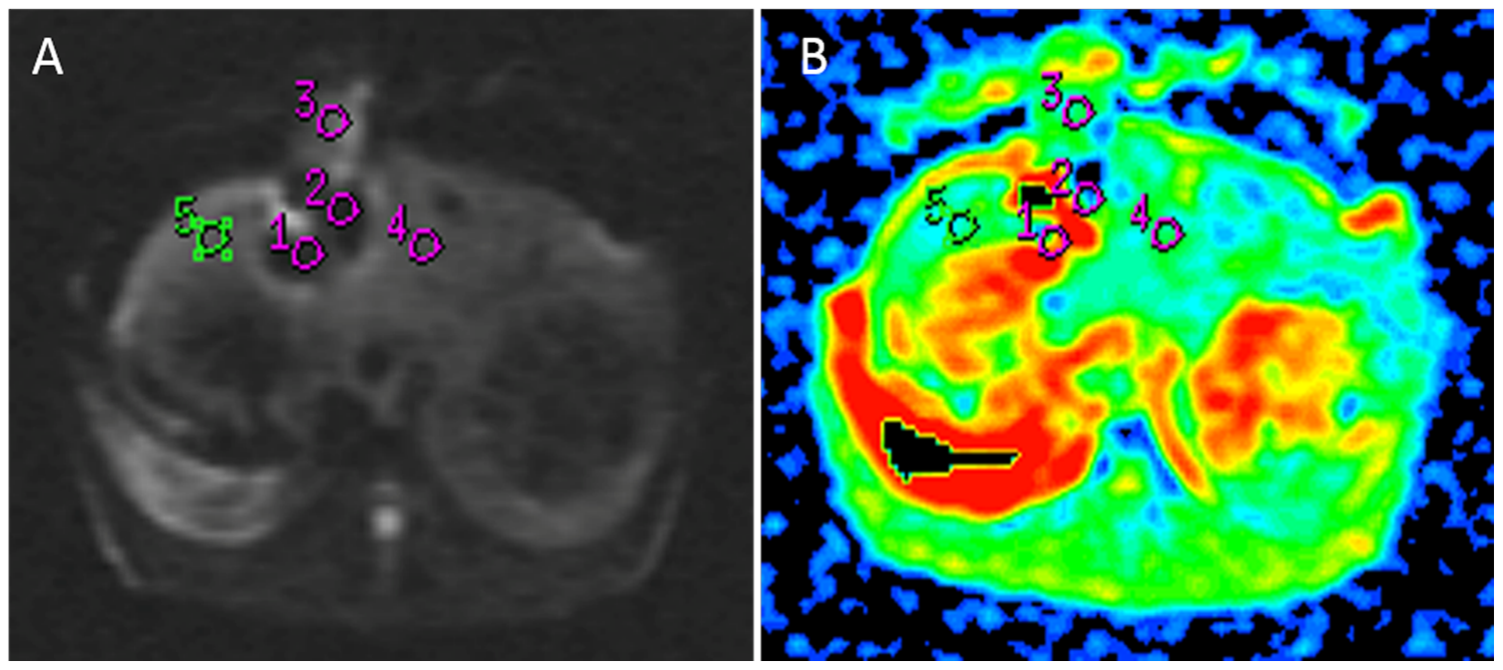
MRI of rabbit liver 7 d after RFA. DWI (A) and ADC map (B) after RFA, hypointensity appeared in necrotic lesion, ring hyperintensity lesion around the necrotic lesion was granulation tissue, edema.

MRI features of the RFA lesions: All RFA lesions were irregular or wedge-shaped 7–10 days after RFA (mean, 8 days). Lesions varied from hypointensity to mild hyperintensity on T1-weighted MR images. Lesion with hyperintensity on T1-weighted image was compatible with hemorrhage. On T2-weighted images, mixed (hyper-, iso-, and hypo-) intensity were observed. Central area of the lesion with hyperintensity on T2-weighted image was consistent with liquefaction necrosis, which occurred more common in larger lesion (Figure 1). Peripheral ring area of the lesion with hyperintensity on T2-weighted image and with remarkable enhancement on contrast-enhanced image were consistent with granulation tissue (Figure 2). Lesion with hypointensity or isointensity within the RFA area on T2-weighted image was consistent with coagulation necrosis. Recurrent tumor appeared as peripheral nodule around the area of RFA, the RFA lesion with irregular contour, isointensity or hyperintensity on T2-weighted image, mild enhancement, and focal ring enhancement around the RFA. Viable tumor showed hypointensity on T2-weighted image. The lesion with hypointensity on T2-weighted image was consistent with edema mixed with granulation tissue.

**Figure 3:**
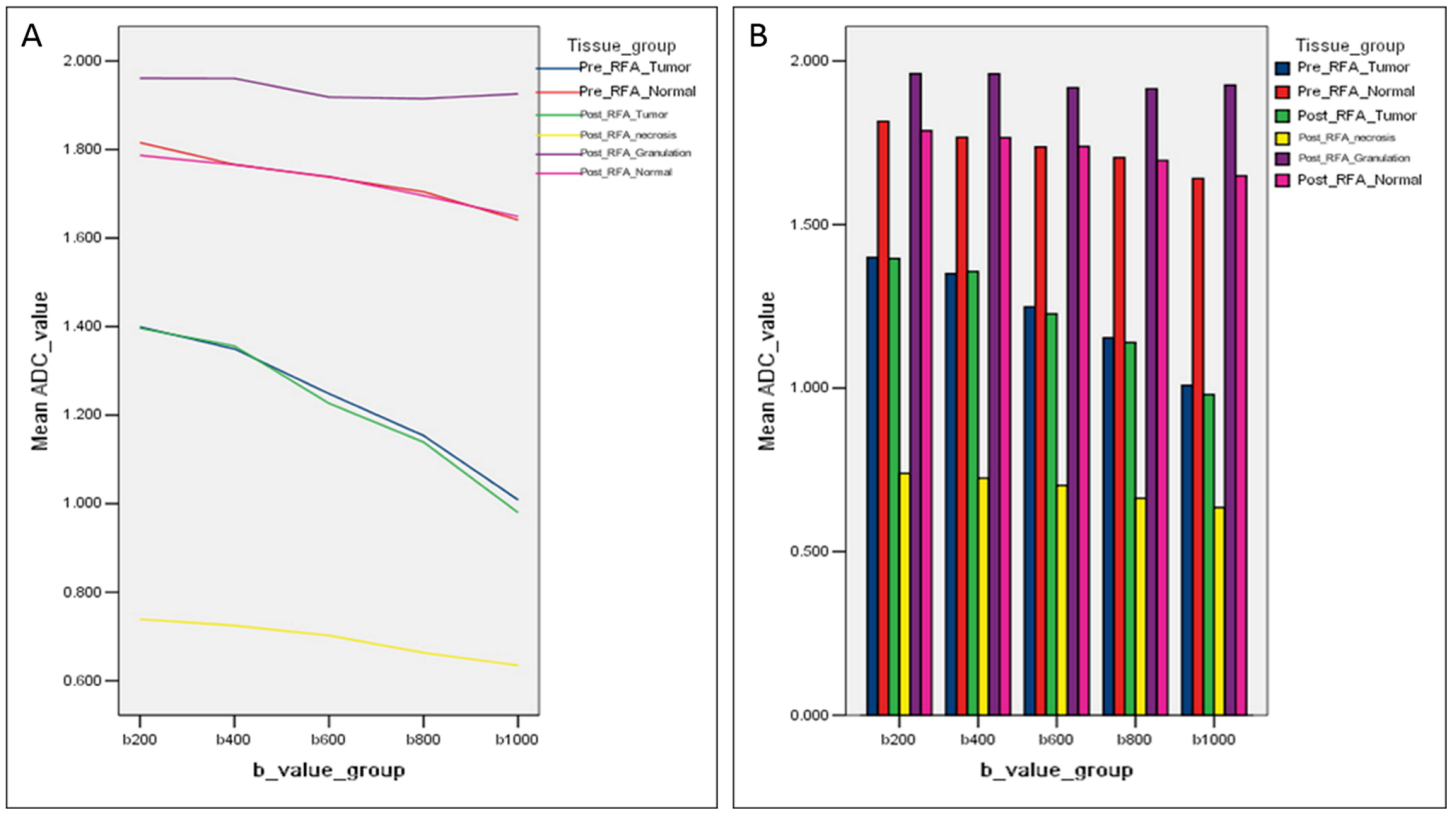
ADC values of different tissues with different b values. Line charts (A), histograms (B) showed that the ADC values of different tissues after RFA from low to high were that of necrosis tissue, viable tumor, normal liver tissue and granulation tissue.

Different ADC values of different tissues with different b values were seen on table 1 and table 2. After RFA, when b was 200, 400, 600, 800, 1000 s/mm^2^, the differences of ADC values among viable tumor, granulation tissue, necrosis, normal liver tissue were significant (*P*<.001). The difference of ADC value between pre- and post-RFA viable tumor was not significant (*P*>.05), the difference of ADC value between pre- and post-RFA normal liver tissue was not significant (*P*>.05). The ADC values of different tissues after RFA from low to high were that of necrosis tissue, viable tumor, normal liver tissue and granulation tissue (Figure 3). In the two non–tumor-bearing animals, capsular retraction and mild enhancement were presented at treatment sites at 1 week, which were sacrificed 1 month after RFA, no histologic evidence of granulation tissue was found.

**Table 1:** ADC values of tumor and normal liver parenchyma with different b values before radiofrequency ablation.

Tissue	N	b = 200	b = 400	b = 600	b = 800	b = 1000
**T**umor	18	1.40±.06 (1.32, 1.48)	1.35±.07 (1.27, 1.43)	1.25±.14 (1.17, 1.32)	1.15±.19 (1.08, 1.23)	1.01±.12 (.93, 1.09)
Normal	18	1.82±.05 (1.78, 2.03)	1.77±.44 (1.59, 1.97)	1.74±.04 (1.63, 1.86)	1.70±.04 (1.56, 1.87)	1.64±.07 (1.49, 1.78)
*P* value		.000	.000	.000	.000	.000

Unit of b value is s/mm^2^, unit of ADC value is 10^−3^ mm^2^/s. The 2 values in the bracket refer to lower bound, upper bound of 95% confidence interval, respectively.

**Table 2:** ADC values of different tissues with different b values after radiofrequency ablation.

Tissue	N	b = 200	b = 400	b = 600	b = 800	b = 1000
**T**umor	9	1.40±.05 (1.29, 1.50)	1.36±.06 (1.25, 1.46)	1.23±.14 (1.12, 1.33)	1.14±.12 (1.03, 1.25)	.98±.08 (.87, 1.09)
**Granulation**	18	1.96±.05 (1.89, 2.04)	1.96±.13 (1.89, 2.04)	1.92±.12 (1.84, 1.99)	1.92±.15 (1.84, 1.99)	1.91±.15 (1.85, 2.00)
N**ecrosis**	18	.74±.04 (0.63, 0.81)	.73±.04 (0.59, 0.86)	.70±.05 (0.60, 0.83)	.66±.05 (0.52, 0.76)	.64±.06 (0.57, 0.72)
**Normal**	18	1.79±.05 (1.68, 1.85)	1.77±.05 (1.64, 1.89)	1.74±.04 (1.59, 1.81)	1.70±.06 (1.53, 1.90)	1.65±.05 (1.46, 1.78)
*P* value		.000	.000	.000	.000	.000

9 of 18 rabbits with viable tumor, and 9 of 18 rabbits with complete necrosis after radiofrequency ablation. Unit of b value is s/mm^2^, unit of ADC value is 10^−3^ mm^2^/s. The 2 values in the bracket refer to lower bound, upper bound of 95% confidence interval, respectively.

Histopathologic findings: When the animals were sacrificed after RFA and MR imaging, local viable tumors were found in 9 (50%) of the 18 treated tumors.

**Figure 4:**
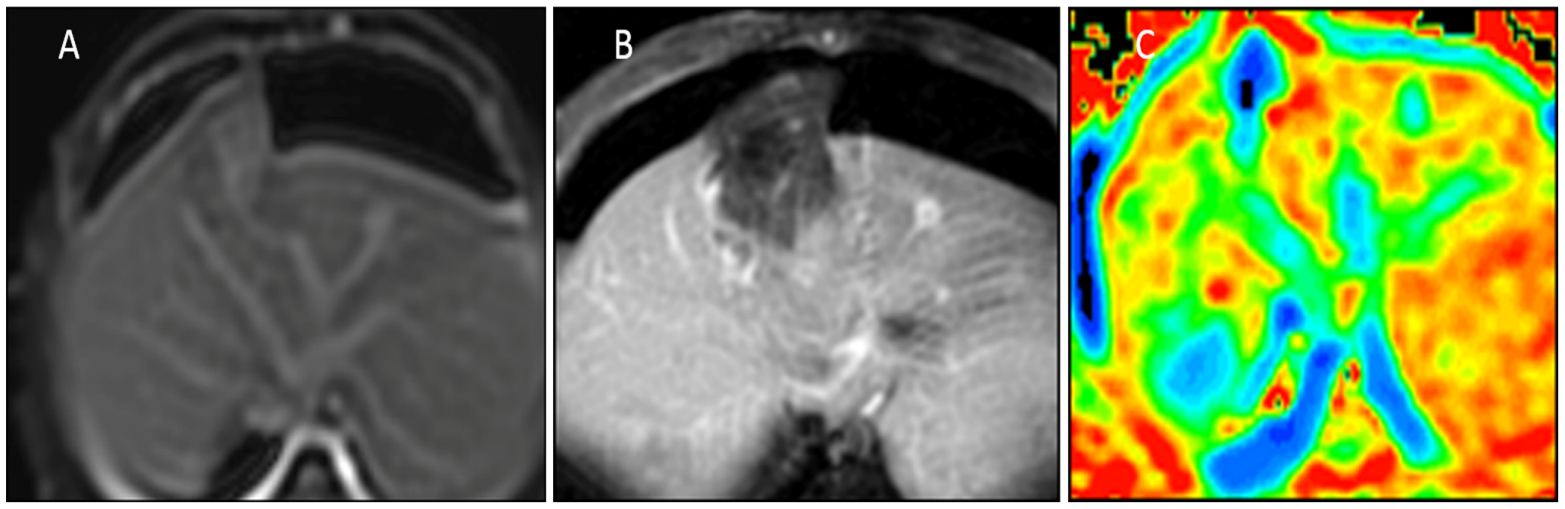
MRI of rabbit liver 7 d after RFA. MR T2WI (A), MR contrast enhancement (B) after RFA, no enhancement appeared in necrotic lesion. DWI ADC map image (C). Complete necrosis after RFA, but the normal crossing vascular within the necrotic tissue was visualized.

Gross specimens of viable tumors were found at the RFA sites in 3 (17%), all 3 viable tumors located at the periphery of RFA. Specimens of viable tumors were gray, microscopic viable tumor cell were round, or oval. Granulation tissue was seen at the interface between viable tumor and adjacent liver parenchyma. Focal nodules were found in 3 viable tumors and measured 5–10 mm in size (mean diameter, 7 mm). There was no embolism occurred in the artery, portal vein, hepatic vein. Histopathologic analysis of the lesion after RFA found that the lesions were mainly consistent with coagulation necrosis. Central liquefaction necrosis was found in larger lesion. Ring granulation tissue, inflammation, or edema measured 1–5 mm was found at the periphery of the lesion. Eight RFA lesions had patent portal venous surrounded by necrosis, with patent hepatic arterial and/or portal venous branches extending into the RFA lesions (Figure 4). In 1 lesion, viable tumor was seen adjacent to portal venous within the necrotic RFA lesion.

## Discussion

In this study we successfully constructed the rabbit liver VX2 tumor models, and the models underwent RFA. We investigated the ADC values of the various tissues after RFA, the ADC values of different tissues after RFA from low to high were that of necrosis tissue, viable tumor, normal liver tissue and granulation tissue. The ADC value of necrotic tissue was remarkably lower than that of the viable tumor tissue. These results were important for the future basic and clinical research of RFA.

The VX2 tumor of the rabbit liver displayed hyperintensity on DWI 14–21 d (mean 17 d) after the implantation of tumor fragments. The ADC value of the tumor was lower than that of the liver parenchyma, secondary to the parenchyma of the tumor containing abundant tumor cells, and the clusters of tumor cells, decreasing signal intensity caused by the restricted diffusion of water molecule was lower than that in the normal liver parenchyma [8], [9].

The physiological function of the animal couldn't completely recover during the early phase after RFA, which would affect the accuracy of the ADC value, so DWI was performed within 7–10 d after RFA [10]. The ADC value of the necrotic tissue decreased after RFA, the pathophysiology mechanism was that dehydration due to carbonization, coagulation necrosis; differences of b value, acquiring sequence and parameters [11]–[13].

The ADC value of the same tissue depending on different b value: the ADC value decreased when higher b value was used [14]. The ADC value was higher when lower b value was used in the same tissue, and the ADC value was more accurate with higher b value, but longer echo time should be used to acquire image with higher b value, and the quality of the image was poorer than that with lower b value [15]–[17]. Therefore, to accurately express the diffusion coefficient with ADC value, and to obtain better image quality, the ideal b value might be chosen as 400–600 s/mm^2^.

The differences of ADC values among viable tumor, granulation tissue, necrosis and normal liver tissue were all significant (*P*<.001). The difference of ADC value between pre- and post-RFA in viable tumor was not significant (*P*>.05), the difference of ADC value between pre and post-RFA in normal liver tissue was not significant (*P*>.05) neither. The pathophysiology mechanism of the decreased ADC value of necrotic tissue was dehydration caused by carbonization, coagulation necrosis. The pathophysiology mechanism of decreased ADC value of viable tumor tissue was that the restricted diffusion of water molecule caused by tumor tissue. The reasons of why the ADC value of granulation tissue and edema was higher than that of the normal liver parenchyma were that the diffusion of water molecule accelerated, caused by the hyperplasia and dilation of the blood capillary surrounding the area of RFA, blood flow rate of the area accelerated, and the blood supply increased [18]–[20].

The mechanisms of local recurrent or residual tumor tissues after RFA and the strategies for decreasing local recurrent or residual tumor tissues were as follows: first, all the local recurrent or residual viable tumors were located at the periphery of RFA, so the location of the probe should be accurately guided by US or CT, and the area of RFA should be larger than that of the tumor, the current ablation strategy attempted to destroy a peripheral 0.5- to 1.0-cm rim of apparently normal tissue surrounding the tumor margin [19]; second, in this study, we found 8 RFA lesions had intact portal venous surrounded by necrosis, with patent hepatic arterial and/or portal venous branches extending into the RFA lesions, in 1 lesion, microscopic viable tumor was adjacent to intact portal venous within necrotic RFA lesion [21]–[24]. The pathophysiology mechanism was that the temperature surrounding the vessel decreased by the flowing blood during RFA [25]–[27], on the other hand, the time of RFA in this study was shorter than that of the clinical application [28], [29]. Granulation tissues were seen in all lesions surrounding the area of the RFA 7–10 d after RFA, but granulation tissues disappeared after 1 month in 2 rabbits of control group. Granulation tissue was seen at the interface between viable tumor and adjacent liver parenchyma in this study. Further study should be performed to verify whether formation of granulation tissue, hyperplasia of capillary, increased angiogenesis can accelerate the growth of residual or recurrent tumor tissue.

This study had several limitations. First, although comparison of pathology smears to ADC maps were strived by repeated measurement in our study, the accuracy between HE smears and diffusion weighted images remained a limitation, more systematic studies of pathology smears correlated with ADC maps may be important to the correlated study. In addition, our purpose was to get the DWI features of various tissues including the viable tumor tissue, so the time of RFA was shorter than that of clinical RFA.

In conclusion, the success rates of constructing the rabbit liver VX2 tumor model and RFA were high, it is important in the basic and clinical research of RFA. MR DWI can be used to follow up the progress of the lesions after RFA, the ADC value of necrotic tissue was remarkably lower than that of the viable tumor tissue, DWI plays an important role in detecting different tissues after RFA, and it is important in the further clinical research.
